# A Strategy for Precise Treatment of Cardiac Malignant Neoplasms

**DOI:** 10.1038/srep46168

**Published:** 2017-04-10

**Authors:** Wenshuo Wang, Jinqiang Shen, Hongyue Tao, Yun Zhao, Hui Nian, Lai Wei, Xiaoyuan Ling, Ye Yang, Limin Xia

**Affiliations:** 1 Department of Cardiac Surgery, Zhongshan Hospital, Fudan University, Shanghai, 200032, P. R. China; 2 Department of Radiology, Huashan Hospital, Fudan University, Shanghai, 200043, P. R. China; 3 Department of General Practice, Pujiang Community Health Service Center, Minhang Districts, Shanghai, 201112, P. R. China

## Abstract

The prevalence of cardiac malignant neoplasms in the general population has been shown to be significant higher than what was previously estimated, yet their treatment has remained difficult and effective therapies are lacking. In the current study, we developed a novel thermotherapy in which PEG-functionalized carbon nanotubes were injected into the tumor regions to assist in the targeted delivery of infrared radiation energy with minimal hyperthermic damage to the surrounding normal tissues. In a mouse model of cardiac malignant neoplasms, the injected carbon nanotubes could rapidly induce coagulative necrosis of tumor tissues when exposed to infrared irradiation. In accordance, the treatment was also found to result in a restoration of heart functions and a concomitant increase of survival rate in mice. Taken together, our carbon nanotube-based thermotherapy successfully addressed the difficulty facing conventional laser ablation methods with regard to off-target thermal injury, and could pave the way for the development of more effective therapies against cardiac malignant neoplasms.

Cardiac malignant neoplasms, including primary and secondary cases, have been suggested by autopsy studies to account for approximately 2.3% individuals in the general population, with the average patient age at around 40 and medium survival at 9 months^1^. Due to the muscular and non-fatty nature of heart, primary heart cancers are relatively rare, comprising only around 0.001–0.03% of the most autopsy cases^2^. Several clinical studies on patients with primary malignant tumors in the heart have all reported sarcoma to be the dominant type^3^,^4^. Meanwhile, secondary cardiac neoplasms, which often originate from lung or breast, have recently been suggested to be far more common than previously thought, with estimated occurrence rates ranging from 0.02 to 14% of the general population^1^,^2^. The metastatic rates varied from 26 to 43% at presentation and 75% at the end of life^2^.

Despite vigorous clinical and academic efforts in search for curative treatment methods, it is still extremely difficult to provide a satisfactory medical solution to patients afflicted with cardiac malignant neoplasms. The cause for this predicament is multifaceted: The difficulty in early detection and diagnosis means that the tumor, when found, has often invaded deep into the myocardium and therefore become impervious to resection. Even if a large portion of the tumor can be removed through surgery, subsequent reconstruction procedures frequently prove to be a daunting, if not completely impossible, task. On the other hand, the use of radiotherapy and chemotherapy for the treatment of cardiac neoplasms is often limited by their side-effects. In fact, cardiotoxicity cases have been reported in numerous clinical studies involving the use of radiation and/or anti-tumor drugs^5^,^6^,^7^. Furthermore, there is evidence suggesting that patients who cannot undergo complete resection often benefit little from such adjuvant treatment methods in the long term^2^. Due to these drawbacks, there is great urgency in developing new cancer therapies that demonstrate higher efficacy and fewer, milder adverse effects.

In recent years, thermotherapy, represented by magnetic resonance imaging (MRI)-guided high-intensity focused ultrasound (HIFU), has emerged as a noninvasive treatment method for solid tumors^3^. For example, Lindner *et al*. applied MRI-guided interstitial photothermal ablation on twelve prostate cancer patients and reported a six-month tumor remission rate of 50% without detectable evidence for perioperative complications^8^. At low-to-moderate intensities (0.125–3 W/cm^2^), ultrasound has been shown to elicit a series of physiological responses on the irradiation zone^4^. In contrast, high-intensity ultrasound beams (5 W/cm^2^) would induce heat-induced coagulative necrosis in the targeted tumor tissues^5^. Compared to surgical ablation, thermotherapy offers several distinct advantages: (i) The noninvasive nature of ultrasound obviates the need of surgical incision, thereby reducing the risk of complications. (ii) The penetrative ability of ultrasound allows access to solid tumors situated at surgically difficult locations. Since thermotherapy requires extreme precision in order to avoid damaging the surrounding healthy tissues, MRI has often been employed to map target regions and monitor tumor margins during treatment^3^. Often, nanomaterials that carry contrast agents are used to enhance MRI signals and provide better scanning resolution^9^,^10^. However, the resolution of MRI is often insufficient for the need of generating full, detailed images of the targeted tumors, necessitating the use of various tumor-specific biomarkers or contrast agents^3^. Furthermore, cardiac cancers are particularly difficult to track by MRI because the periodic contraction of heart muscles makes imaging and focusing of the ultrasound beams virtually impractical.

To address the technological difficulties of thermotherapy in treating cardiac malignant neoplasms, we reasoned that instead of relying on high-resolution images for aiming, a tumor-specific heat director could be used to focus thermal energy on areas with cancerous tissues. The ability of carbon nanotubes to absorb infrared radiation^6^,^7^ and their amenability to various covalent functionalization methods make them particularly well-suited for this purpose^11^. Herein we report a novel strategy that applies carbon nanotube-directed infrared radiation to the treatment of cardiac malignant neoplasms. To the best of our knowledge, our study is the first demonstration of using thermotherapy for the treatment of deep-seated and moving tumors.

## Methods and Materials

### Cell culture

A human lung cancer cell line NCI-H460 transfected with firefly luciferase reporter gene was cultured in Dulbecco Modified Eagle Medium (DMEM, Thermo Fisher Scientific, Waltham, MA, USA) supplemented with 10% fetal bovine serum (FBS, Thermo Fisher Scientific, Waltham, MA, USA). Cells were grown to the logarithmic phase and harvested for use in the subsequent assays.

### Carbon nanotubes synthesis and characterization

The synthesis of single-walled carbon nanotubes was performed on a quartz tube furnace (Lindberg Blue, Thermo Fisher Scientific, Waltham, MA, USA). To generate water vapor, a carrier gas was passed through a water bubbler filled with ultrapure water. The concentration of the generated water vapor was monitored by a water sensor installed in the exhaust tunnel. The carrier gas consisted of 60% of Ar (99.9999%) and 40% of H_2_ (99.9999%). The total flow of the carrier gas was 1000 cm^3^ per minute under standard temperature and pressure. Carbon nanotubes were grown at 750 °C using pure ethylene (99.9999%) as the carbon source under the catalysis of Al_2_O_3_/Fe. The formed carbon nanotubes were harvested by a razor blade and suspended in pure alcohol by ultrasonic disruption. Polyethylene glycol 1000 (PEG, Merck, Darmstadt, Germany) was added to the suspension to a final concentration of 5% in order to modify the surface of the carbon nanotubes to achieve better hydrophilicity. The structure of the prepared carbon nanotubes was characterized by transmission electron microscopy (TEM) (CM 200, Philips, Amsterdam, Netherland) and the size distribution was determined by a laser particle analyzer (Mastersizer 3000, Malvern, Worcestershire, UK).

### Cell viability analysis

NCI-H460 cells were divided into three experimental groups, the carbon nanotube group, the laser group and the control group. For cell viability analysis, both the carbon nanotube group and the laser group were incubated with 100 μg/mL of carbon nanotubes in DMEM for 24 h. After incubation, the cells in the laser group were subjected to laser treatment at an intensity of 0.5 W/cm^2^ for 2 min. In contrast, the control group was neither incubated with carbon nanotubes nor exposed to laser. The viable cells in each group were quantified using a Cell Counting Kit-8 (Dojindo Molecular Technologies, Rockville, MD, USA) following the manufacturer’s instructions.

### Uptake Assay

FITC-conjugated PEG was used instead of regular PEG to obtain fluorescent carbon nanotubes, following the same synthetic procedures described above. NCI-H460 cells were incubated with 50 μg/mL of the fluorescent carbon nanotubes in DMEM supplemented with 10% FBS. After four hours of cultivation, the medium was removed and the cells were washed by PBS twice. After the addition of fresh complete medium, the cells were imaged under a confocal microscope (FV1200, Olympus, Shinjuku, Tokyo, Japan).

### Animal model

According to the guidelines approved by the Ethics Committee of Zhongshan Hospital, BALB/c (nu/nu) nude mice were anesthetized intraperitoneally with ketamine at a dose of 90 mg/kg (Merck, Darmstadt, Germany) and xylazine at 8 mg/kg (Merck, Darmstadt, Germany), and placed on a thermostat pad. The mice were then subjected to an endotracheal intubation procedure and connected to a ventilator, followed by thoracotomy to expose the heart. A sterile precision syringe (Nanofil 100 μL syringe, World Precision Instruments, Sarasota, FL, USA) casted with a 34G needle (World Precision Instruments, Sarasota, FL, USA) was used to deliver 10^6^ NCI-H460 cells into the right ventricular free wall. The thoracic wall was closed, followed by two days of recovery support. The formation of cardiac neoplasms was confirmed by echocardiography in seven days.

### Echocardiography

Mice were anesthetized by inhalation of 1% isoflurane (Baxter, Deerfieled, IL, US). Each subject was fastened to a heating pad and the chest was shaved using a chemical hair remover (VEET, Shanghai, China). Pre-warmed ultrasound gel (Hankang, Hangzhou, Zhejiang, China) was applied to the thoracic wall to enhance the visibility of the heart chambers. M-mode and two-dimensional echocardiographic examinations were performed on a Vevo 2100 high-resolution ultrasound imaging system (FUJIFILM VisualSonics, Toronto, Canada).

### *In vivo* photothermal therapy

A of 100 tumor-free BALB/c (nu/nu) nude mice were used to determine the safe temperature range in the hyperthermia therapy. The mice were subjected to an intramyocardial injection of 6 μL normal saline containing 6 μg carbon nanotubes and then divided equally into ten groups, each of which was assigned with a target treatment temperature (from 41 °C to 50 °C). The temperature was measured by a thermal camera(A325sc, FLIR Systems Inc, Wilsonville, OR, US). The survival rate of each group was monitored daily over a one-month period following the irradiation.

In order to demonstrate the efficacy of carbon nanotubes in laser-induced thermal therapy, a total of twelve tumor-bearing mice were equally divided into the treatment group and the control group. The mice in the treatment group underwent reoperation to inject the neoplasm with 6 μL of normal saline containing 6 μg carbon nanotubes. The control group was injected with 6 μL of normal saline. The anesthetized mice were exposed to a near-infrared laser beam at an intensity of 1.5 W/cm^2^ for 2 min. The output power of the laser was adjusted to ensure that the temperature of the neoplasm was maintained in the previously determined safe range. The neoplasm size and the cardiac functions of the mice in both groups were measured every week and normalized to their initial values. The NCI-H460 cell load was monitored by bioluminescence over a period of one month. Mice with left ventricular ejection fraction below 40% were humanly sacrificed.

### Histological staining

Hearts were harvested from tumor-bearing mice (with and without laser treatment) and fixed in 4% paraformaldehyde for 24 hours. After fixation, the heart was embedded in paraffin and cut into 7 μm-thick slices. The heart tissue sections were subsequently mounted on slides and stained with hematoxylin and eosin. Images were acquired randomly at 40X magnification under a light microscope (FV1000, Olympus, Shinjuku, Tokyo, Japan).

### Statistical analysis

The data were presented as mean ± standard deviation. Time courses were compared using the variance analysis of repetitive measurements. Group differences were evaluated by student t-test. P < 0.05 was considered statistically significant.

## Results

### PEG modified single-walled carbon nanotube exhibited good biocompatibility and cytotoxicity effect

**Figure 1:**
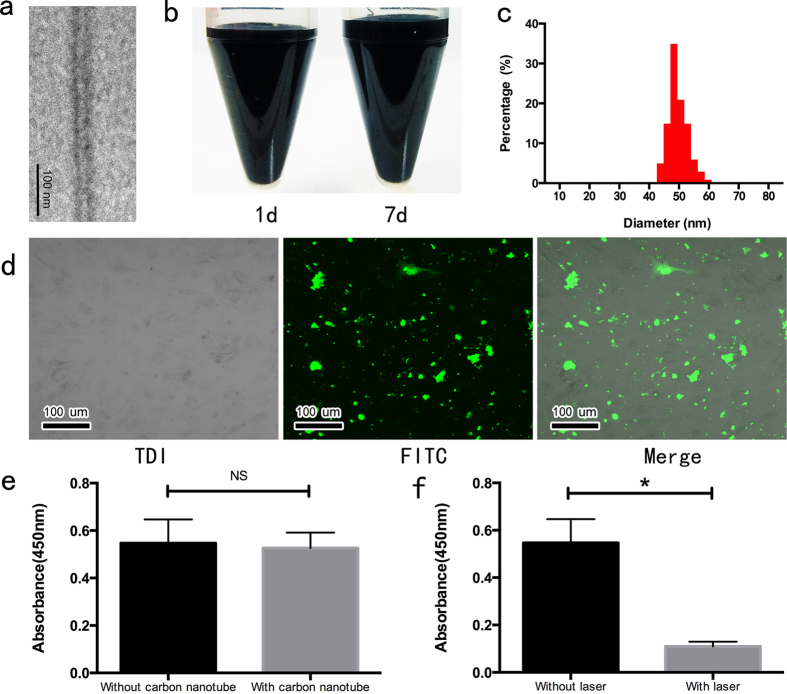
Structural and functional characterization of single-walled carbon nanotubes. (**a**) TEM image of the synthesized carbon nanotubes; (**b**) Dispersion of carbon nanotubes in a stable aqueous suspension; (**c**) Size distribution of the carbon nanotubes as determined by a laser particle analyzer; (**d**) Internalization of green-fluorescent carbon nanotubes by NCI-H460 cells; (**e**) Carbon nanotubes exhibited no detectable cytotoxicity in the absence of laser treatment; (**f**) Exposure of carbon nanotube-treated cells to laser irradiation significantly reduced the number of viable cells. ***Abbreviation***: TDI, transmitted detector integration; FITC, fluorescein isothiocyanate.

The synthesized carbon nanotubes were indicated by TEM imaging to comprise a single layer of graphene wall (Fig. 1a). PEG modification was found to significantly improve the dispersion and reduce the aggregation tendency of the carbon nanotubes in aqueous solution over the 7-day observation period (Fig. 1b). After phacofragmentation and surface modification, the final average size of the carbon nanotubes was approximately 50 nm (Fig. 1c). The uptake assay was performed by incubating FITC/PEG-conjugated carbon nanotubes with NCI-H460, a human lung cancer cell line, and subsequently visualizing cell distribution via confocal microscopy. The images confirmed that the nanotubes were mostly internalized or strongly adhered to the cell surface within four hours following the incubation (Fig. 1d). We next performed cell viability assays using CCK-8 kit to examine the anti-cancer effect of laser irradiation and carbon nanotubes *in vitro*. As illustrated in Fig. 1e, incubating the cells with carbon nanotubes in the absence of laser exposure did not induce apoptosis (P = 0.021). On the other hand, the cancer cells incubated with carbon nanotubes showed reduced viability only when subjected to laser treatment, as indicated by the sharp decrease of fluorescence at 450 nm. (P = 0.021, Fig. 1f).

### PEG-modified single-walled carbon nanotubes increased tissue temperature around the injection site

**Figure 2:**
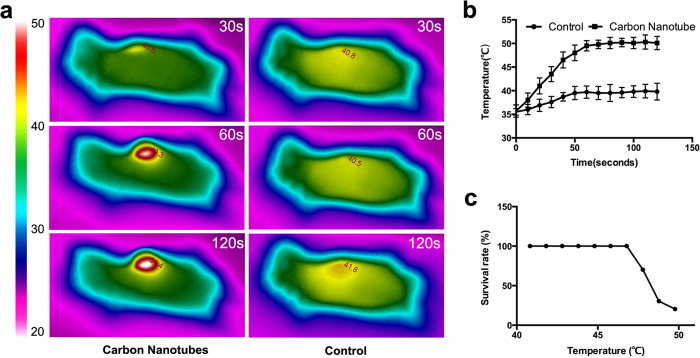
The determination of safe temperature range. (**a**) Whole-body imaging by thermal camera showed that the temperature of the impacted cardiac tissues in the mice injected with carbon nanotubes (left) was significantly higher than that in the control (right); (**b**) Time course of temperature increase in the irradiation zone for both the treatment group and the control group; (**c**) The two-month survival rates at treatment temperatures from 41 to 50 °C.

Whole-body imaging by thermal camera confirmed that the injected carbon nanotubes could efficiently absorb infrared radiation and convert its energy to heat, which was subsequently delivered to the neighboring tissues through thermal conduction leading to local hyperthermia (Fig. 2a). The temperature of the impacted murine heart tissues was linearly increased to around 50 °C within 60 s as a result of the laser treatment when carbon nanotubes were administered. In contrast, the control group showed a much smaller increase of temperature around the irradiated region (P = 0.014, Fig. 2b & Table S1). The two-month survival rate for the treatment group began to decline once the target temperature of the thermotherapy exceeded 48 °C (Fig. 2c). Taken together, we concluded that the acceptable range of target temperature for treating cardiac neoplasms should be from 41 to 47 °C.

### Carbon nanotubes guided thermotherapy alleviated tumor load in mice hearts

**Figure 3:**
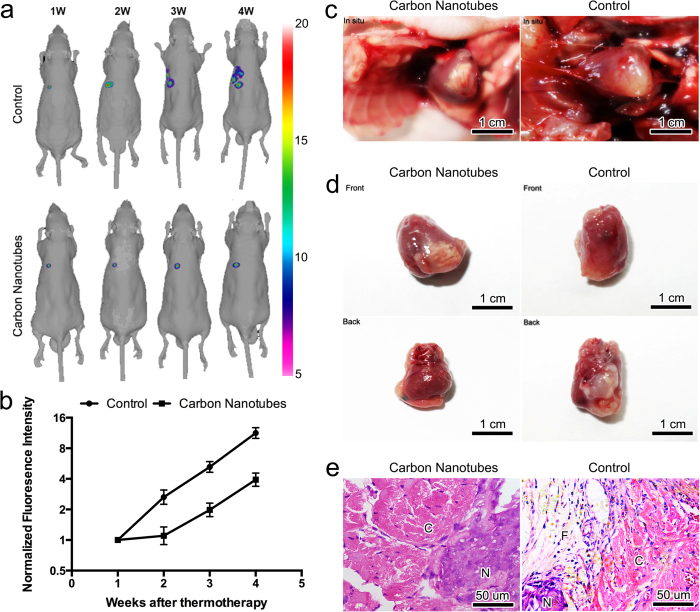
Thermotherapy can effectively reduce tumor load in mice injected with carbon nanotubes. (**a**) Bioluminescence imaging results demonstrating that tumor growth was significantly inhibited in the treatment group that received injection of carbon nanotubes in the heart in comparison to the control group; (**b**) Semi-log plot showing the time course of tumor proliferation as indicated by normalized fluorescence intensity in both the treatment group and the control group; (**c**) Chest autopsy of tumor-bearing mice. Compared to the control group, mice injected with carbon nanotubes showed strong evidence of coagulative tissue necrosis after laser treatment; (**d**) Heart autopsy results; (**e**) Histological examination of neoplasm in the dissected heart specimens. C: myocardial tissues; N: malignant neoplasms; F: fibrous tissues.

Tumor load is generally regarded as one of the most important prognostic factors for cancer patients. *In vivo* bioluminescence imaging indicated that compared to the control, tumor growth and proliferation was significantly suppressed in the treatment group (P < 0.001, Fig. 3a,b & Table S2). This was consistent with the autopsy results that showed extensive tumor invasion into the left ventricle wall in the control group (Fig. 3c). Furthermore, fibrous legions were clearly visible in the irradiation zone, which are signs of thermally induced coagulative necrosis of myocardial tissues (Fig. 3d). Histological examinations provided evidence that the infiltrated tumor cells were replaced by fibroblasts with prominent collagen bands in between (Fig. 3e).

### Carbon nanotubes guided thermotherapy alleviated the deterioration of cardiac function and improved the survival rate in tumor-bearing mice injected with carbon nanotubes

**Figure 4:**
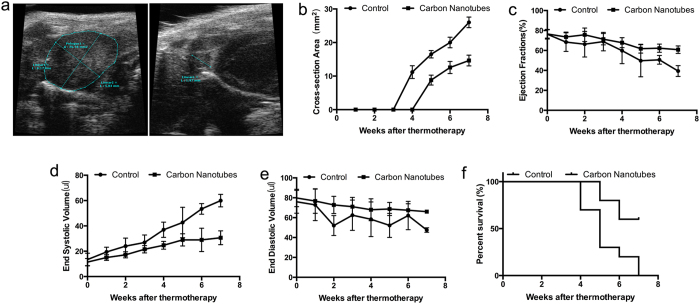
Thermotherapy alleviated the deterioration of cardiac function in mice that received injection of carbon nanotubes. (**a**) Echocardiographic examination showing the representative signs of neoplasms (left: control, right: carbon nanotubes); (**b–e**) Comparison of tumor cross-section areas (**b**), ejection fractions (**c**), end-systolic volumes (**d**) and end-diastolic volumes (**e**) between the treatment group and the control group; (**f**) The survival curves for the treatment group and the control group.

The infiltration and migration of cardiac malignant neoplasms frequently lead to reduced heart function. Echocardiographic examinations showed that the average cross-section area of the heart tumors in mice administered with carbon nanotubes increased at a substantially lower rate compared to that in the control (P < 0.0001, Fig. 4a,b). This is mirrored by the finding that key cardiac function parameters, including ejection fraction (EF) and end systolic volume (ESV), exhibited significantly slower decline (P = 0.027 and 0.016, respectively, Fig. 4c,d) in the treatment group compared to the control group. However, no statistically significant difference in end diastolic volume (EDV) was observed between the two experiment groups (P = 0.059, Fig. 4e). In addition, the overall survival rate of the treatment group was substantially higher than that of the control group, and even remained above 50% seven weeks after the laser ablation, at which point no animal subject from the control group was still alive (P = 0.0096, Fig. 3f).

## Discussion

Laser-induced hyperthermia exerts cytotoxic effects on cancerous tissues via multiple mechanisms. On the one hand, high-energy laser beams can cause local destruction of the cancerous tissues on which they are focused, a process commonly referred to as coagulative necrosis. Studies have shown that heat-induced cell injuries consist of direct damage to various cellular components immediately following the laser application, and indirect damage that occurs progressively over a sustained period of time involving apoptosis, microvascular damage and other alterations of essential cellular functions^12^. Temperatures exceeding 60 °C can rapidly denature protein and destroy the structural integrity of cell membranes, effectively leading to near-instantaneous cell death^12^. In addition, elevation of temperature has also been associated with increased proton permeability across the inner mitochondrial membrane in hepatocytes^13^. On the other hand, there is also increasing evidence that laser ablation can elicit active immune response in the exposed tumor tissues. Kallio *et al*.’s study of 13 liver cancer patients found that thermal ablation stimulated an early immune response by up-regulating the serum levels of interleukin-6 and tumor necrosis factor-α receptor^14^. Similarly, patients with metastasized colorectal malignancies exhibited significant stimulation of CD3+, CD4+ and CD8+ cells after laser treatment, signifying a tumor-specific cytotoxic T cell activation^15^. Due to these effects, laser-induced thermal ablation has been increasingly explored as a promising therapeutic method for treating malignant tumors, particularly those that are located at surgically inaccessible or difficult positions.

Despite their relatively low rate of occurrence, cardiac malignant neoplasms are notoriously resistant to nearly all forms of conventional treatment approaches. Surgical removal is often thwarted by difficulties in preserving vital organ structures of the heart, while chemotherapy and radiotherapy are generally precluded due to their pronounced cardiotoxicity. Although there is no report thermal ablation for the treatment of cardiac malignant neoplasms up to this date, this technique has been proposed and tested for other heart-related anomalies. For instance, radiofrequency catheter ablation is a common treatment approach for atrial fibrillation^16^,^17^. The major problem that faced surgeons in these procedures is that cardiac motility often led to unintended thermal damage to the proximal esophageal tissues. Heart motions are also a serious concern for thermal therapies performed for liver cancer^18^. To address the problem that MRI and other image-guided methods are poorly-suited by nature for monitoring non-static tumors, including cardiac malignant neoplasms, we chose PEG-functionalized carbon nanotubes to assist with the targeted delivery of radiation energy in the current study. Carbon nanotubes possess unique photothermal properties such as excellent thermal and electrical conductivity^19^. Because of this, Gannon *et al*. hypothesized that they could serve as suitable “heat sinks” that induce hyperthermia in cancerous tissues by remotely absorbing electromagnetic and subsequently converting it to heat^7^. Indeed, by using carbon nanotube-assisted radiofrequency radiation, the authors achieved complete necrosis of liver tumors in a rabbit model. In another study, Nyugen and colleagues argued that the internalization of carbon nanotubes could modify the thermal conductivity of the affected tissues, which amplified the cytotoxic effect of the laser ablation^20^. Consistent with these results, our laser irradiation of cardiac malignant neoplasms infiltrated with carbon nanotubes drastically decreased their volumes (by 30–40%) in nude mice within two weeks of the treatment. The autopsy results indicated that hyperthermic necrosis was localized in the tumor region and no significant damage of the normal cardiac tissues was observed. In addition to heat conduction, carbon nanotubes are also amenable to chemical modifications by functional moieties that range from fluorophores^21^ and polymers^22^ to antibodies^23^, which can enhance their detectability, biocompatibility and/or target-specificity. Indeed, we confirmed in the current study that the PEG-conjugated carbon nanotubes showed significant reduction in aggregation and improvement in biocompatibility compared to their unmodified counterparts. It should be stressed that the cytotoxicity of carbon nanotubes remains an unresolved issue, with the predominant majority of studies suggesting mild or no cytotoxicity^24^. Here in this study, our viable cell analysis, which the safe concentration of 100 μg/mL, provided valuable experimental results that added to the current pool of data on the potential safety impact of carbon nanotubes as a heat inducer and/or delivery system. We then extended our toxicity evaluation to mouse models by injecting carbon nanotubes at the common dose for treatment, even in the condition of laser radiation, and also found no significant adverse effect on major organs over two months even at these level.

Our pre-clinical results based on the murine model of cardiac malignant neoplasm are comparable with similar *in vivo* studies of carbon nanotubes performed on other types of malignant tumors. In Ding *et al*.’s study, nude mice injected with human breast adenocarcinoma cells were used as the cancer model. After the tumor had grown to 6 mm in diameter, intratumoral administration of 100 μg iron-containing multi-walled carbon nanotubes was shown to induce a >90% reduction of the tumor volume 14 days following laser irradiation^25^. In another study, using antibody-conjugated carbon nanotubes in conjunction with laser ablation, Wang *et al*. successfully shrunk the size of xenotransplanted glioblastoma in nude mice by around 60% within two weeks and achieved near-complete tumor destruction within four to five weeks^26^. Nevertheless, the nanotubes were pre-incubated with the cancerous cells prior to xenografting, and no experiment was conducted to study the efficacy of the intratumoral injection method. Overall, the predominant majority of studies on the use of carbon nanotubes in thermal therapy of malignant tumors were performed *in vitro*, whereas very few offered strong *in vivo* evidence in an experiment context that closely resembled real clinical applications. In this regard, our data obtained from the murine model not only constituted the first of its kind for the treatment of cardiac malignant neoplasms, but also improved the understanding in general of the clinical feasibility of using carbon nanotubes in thermal therapy against cancers. It should be pointed out, however, that our procedures did not result in significant changes in the doubling time of the tumor cells. We speculate that this could have been caused by the fact that the cells were treated for no more than 1 min. In comparison, similar thermal ablation studies that successfully increased the doubling time of tumor cells by a significant margin generally involved longer treatment periods. For instance, in Zölzer *et al*.’s experiments, the doubling time of human melanoma cells was shown to have almost tripled following an one-hour thermal treatment at 43 °C^27^. Further method optimization would undoubtedly lead to improved anti-tumor effect of our phototherapy.

In summary, we have reported the first case of using PEG-functionalized carbon nanotubes in combination with laser-based thermotherapy against cardiac malignant neoplasms. We demonstrated that our carbon nanotubes could be facilely internalized by tumor cells and rapidly induce apoptosis only when laser irradiation was applied. Furthermore, injection of the functionalized carbon nanotubes into the cardiac malignant neoplasms of mice, followed by laser ablation treatment, resulted in rapid induction of hyperthermia in the irradiation zone. By carefully manipulating the target treatment temperature, we achieved coagulative necrosis of the tumor tissues with minimal damage to the surrounding healthy tissues, which was accompanied by the restoration of heart functions in mice. Taken together, our study could potentially lead to the development of more effective therapies for treating cardiac malignant neoplasms.

## Additional Information

**How to cite this article**: Wang, W. *et al*. A Strategy for Precise Treatment of Cardiac Malignant Neoplasms. *Sci. Rep.* **7**, 46168; doi: 10.1038/srep46168 (2017).

**Publisher's note:** Springer Nature remains neutral with regard to jurisdictional claims in published maps and institutional affiliations.

## Supplementary Material

Supplementary Figures
