# Supplementary material for: A Strategy for Precise Treatment of Cardiac Malignant Neoplasms
Source: Sci Rep. 2017 Apr 10;7:46168. doi: 10.1038/srep46168 (PMC5385561; doi:10.1038/srep46168)
Supplement: Supplementary Figures [file srep46168-s1.doc]

**A Strategy for Precise Treatment of Cardiac Malignant Neoplasms**

Wenshuo Wang^1,†^, Jinqiang Shen^1,†^, Hongyue Tao^2,†^, Yun Zhao^1^, Hui Nian^1^,

Lai Wei^1^, Xiaoyuan Ling^3^, Ye Yang^1,*^, Limin Xia^1,*^

1. Department of Cardiac Surgery, Zhongshan Hospital, Fudan University, Shanghai, 200032, P.R. China

2. Department of Radiology, Huashan Hospital, Fudan University, Shanghai, 200043, P.R. China

3. Department of General Practice, Pujiang Community Health Service Center, Minhang Districts, Shanghai, 201112, P.R. China

† These authors contribute equally to this study.

Corresponding authors:

**Professor Limin Xia**

Room 643, Building No.16, No.1609 Xietu Road, Xuhui Districts, Shanghai, 200032, P.R. China

Telephone Number :+86-21-64041990

Fax Number :+86-21-64041990

Email: 406495702@qq.com

**Doctor Ye Yang**

Room 633, Building No.16, No.1609 Xietu Road, Xuhui Districts, Shanghai, 200032, P.R. China

Telephone Number :+86-21-64041990

Fax Number :+86-21-64041990

Email: yang.ye@zs-hospital.sh.cn

**Table S1. The raw data for temperature curve in Figure 2b**

| Time (s) | Contol (℃) | | | Carbon Nanotubes (℃) | | |
| --- | --- | --- | --- | --- | --- | --- |
| 0 | 35.4 | 35.9 | 35.2 | 36.6 | 34.1 | 36.5 |
| 10 | 36.9 | 35.9 | 35.2 | 38.6 | 37.4 | 38.1 |
| 20 | 36.9 | 37.2 | 36.5 | 42.1 | 38.5 | 42.5 |
| 30 | 36.6 | 38.6 | 37.8 | 43.8 | 42.8 | 43.8 |
| 40 | 38.8 | 38.6 | 38.6 | 45.9 | 45.8 | 47.7 |
| 50 | 39.2 | 40.7 | 38.6 | 47.9 | 48.9 | 47.6 |
| 60 | 39.9 | 40.7 | 38.7 | 49.7 | 49.8 | 48.6 |
| 70 | 38.9 | 41.1 | 38.9 | 49.9 | 49.3 | 50.5 |
| 80 | 39.1 | 39.9 | 39.4 | 50.5 | 49.5 | 50.2 |
| 90 | 38.9 | 39.9 | 39.9 | 49.9 | 50.1 | 50.6 |
| 100 | 39.2 | 40.2 | 40.1 | 50.2 | 49.8 | 50.4 |
| 110 | 38.9 | 40.8 | 39.9 | 50.2 | 50.5 | 50.4 |
| 120 | 39.4 | 40.1 | 39.6 | 50.2 | 49.4 | 50.8 |

**Table S2. The normalized raw data for fluorescent intensity in Figure 3b**

| Time (week) | 1 | 2 | 3 | 4 |
| --- | --- | --- | --- | --- |
| Control | 1 | 2.5 | 4.4 | 9 |
|  | 1 | 1.9 | 4.9 | 11.2 |
|  | 1 | 2.9 | 5.1 | 12.9 |
|  | 1 | 3.1 | 5.7 | 11.3 |
|  | 1 | 2.8 | 6.1 | 12.1 |
|  | 1 | 2.6 | 5.3 | 11.9 |
| Carbon Nanotubes | 1 | 1.2 | 2.1 | 4.3 |
|  | 1 | 0.9 | 1.7 | 3.9 |
|  | 1 | 1.4 | 2.1 | 2.9 |
|  | 1 | 1.2 | 2.4 | 4.6 |
|  | 1 | 0.8 | 1.6 | 3.9 |
|  | 1 | 1.1 | 1.9 | 4.5 |
